# A longitudinal investigation on problematic Facebook use, psychological distress and well-being during the second wave of COVID-19 pandemic

**DOI:** 10.1038/s41598-022-26281-0

**Published:** 2022-12-17

**Authors:** Rubinia Celeste Bonfanti, Laura Salerno, Agostino Brugnera, Gianluca Lo Coco

**Affiliations:** 1 Department of Psychology, Educational Science and Human Movement, University of Palermo, Viale delle Scienze, Edificio 15, 90128 Palermo, Italy; 2 Department of Human and Social Sciences, University of Bergamo, Bergamo, Italy

**Keywords:** Psychology, Health care

## Abstract

The social isolation and the subsequent, increased use of Social Networking Sites due to the COVID-19 pandemic have had an impact on subjective well-being around the world. The present longitudinal study examined whether changes in psychological distress and well-being during the Italian second wave of the pandemic differ among people with different levels of Problematic Facebook Use (PFU). A total of 493 participants (M_age_ = 24.55 ± 7.25; 80.3% females) completed measures of passive use of Facebook, social comparison orientation on Facebook, fear of missing out, psychological distress (depressive symptoms and fear of COVID-19 pandemic) and well-being across three waves. Latent class analysis (LCA) categorized participants into three groups with different PFU levels: healthy users, moderate PFU users, and high PFU users. Hierarchical linear modeling (HLM) showed that the between-person level (class membership) accounted for most of the variability in psychological distress and well-being. No significant changes were found in psychological distress and well-being over time, but the High PFU users showed greater levels of psychological distress and lower levels of well-being at each time point. The findings of this study suggest that the relationship between PFU, psychological distress and well-being may reflect trait-like time-invariant differences between individuals rather than state-like changes.

## Introduction

The COVID-19 pandemic enhanced the individual’s use of social media and increased the risk of acquiring addictive tendencies^1^. In the last few years, the restrictions aimed at lessening the spread of the virus have resulted in social distancing, curfews, and shelter-in-place orders across the globe, all of which has led to limited interpersonal and close relationships. During this difficult time, people were overwhelmed by the continual desire to stay connected with others and improve interpersonal communication, and this need was easily satisfied by using social media, such as Facebook^2,3^. However, there is a debate on the consequences of heavy Facebook use for an individual’s well-being^4,5^, and whether problematic Facebook use (PFU), defined as a lack of self-regulation in one’s own use of Facebook, leading to problems in the user’s life^6,7^, can be conceptualized as dysfunctional behavior. Although Facebook might prove valuable in enhancing social contact, receiving positive feedback (there is no dislike button on this platform) and by enhancing social capital^8,9^, PFU is considered as a dysfunctional use of Facebook; it has been related to clinical impairments in various areas of one’s life, such as increased psychological distress and sense of loneliness, decreased self-esteem and life satisfaction^10–13^. Although PFU is associated with time spent online^14^, frequency of Facebook use does not seem to capture the core issues related to PFU^15^. Prior research adopted the broader construct of Problematic social media use (PSMU), which refers to an enduring preoccupation with social media (e.g., Facebook, Instagram, Tik-Tok) that can lead to impairment in well-being^16^ and/or nervousness, loneliness and other psychological problems^17^. However, focusing on specific social media platforms, such as Facebook, more than broad umbrella-constructs can be helpful to understand this complex phenomenon in more depth.

Previous meta-analytic evidence showed that PFU is associated with excessive or problematic Internet use^15^, but probably boasting distinctive features. For example, the hypothesis for passive social media use (e.g., scrolling through news feeds or looking at other users’ profiles, without engaging in direct social interactions) posits that passive Facebook use can lead to a decline in well-being^18,19^. Recent reviews and meta-analytic evidence, from both cross-sectional and longitudinal studies, supported a negative association between passive social media use (i.e., content consumption, browsing with low social connection) and well-being outcomes^4,5,20^.

Passive Facebook use might bring about negative feelings or distress because it may induce upward social comparison regarding dimensions that are important to one’s self-worth and social connectivity^21,22^. Contents posted by others are usually positively skewed, and social comparison can make readers feel negative about their own lives^23,24^. The meta-analysis by Yoon and colleagues^25^ showed that social comparisons on Facebook were more strongly related to depression than was the time actually spent, suggesting that an extensive use of SNSs might lead individuals to compare themselves with other users in a negative way, thus resulting in lower subjective well-being.

Prior research suggested that Fear of Missing Out (FoMO; i.e., “a pervasive apprehension that others might be having rewarding experiences from which one is absent”, [26; p. 1841] may also represent a reinforcement mechanism of PFU^27^. Problematic Facebook users experiencing unwanted feelings or a sense of loneliness when they use Facebook might report increased levels of FoMO^2,28,29^. Specifically, individuals who are afraid of being excluded from the world of Facebook and who are in situations of physical isolation might increase widespread use or PFU^30^.

To sum up, previous research suggested that the mental health consequences of SNSs such as Facebook may critically depend on the way they are used^8,31^. PFU encompasses different domains (e.g., passive use, social comparison, FoMO) which are related to the individual’s need for relatedness, which may lead to lower well-being or distress. However, early research into PFU-well-being effects relied primarily on cross-sectional data^6^ and did not take into account how these effects varied in magnitude between individuals^32,33^. Thus, examining how PFU characteristics co-occur may provide a valuable research option. Prior research has utilized clustering techniques to identify distinct groups and patterns of problematic social media use^34–37^. In the current study we examine different patterns of PFU by Latent Class analysis (LCA) and whether these empirically-derived subgroups differ on both well-being and psychological distress over time. LCA derives a set of latent variables from a series of observed variables and allocates them to a latent class. Thus, this method can be useful in exploring the multifaceted nature of PFU and revealing its maladaptive patterns.

In the present investigation we focus on the link between patterns of PFU and well-being during the COVID-19 outbreak, which has thus far not received extensive research attention. Although people have been using Facebook heavily for sharing COVID-19 information^38^, prior studies showed an association between problematic social media or passive use, online social comparison, FoMO with different facets of an individual’s distress during the first wave of the pandemic^1,2,22,32,39,40^. As previously mentioned, PFU takes place when the individual is characterized by a lack of self-regulation in one’s use, with negative consequences in daily activities, social relationships, and psychological health^15^. During the pandemic, problematic Facebook use appeared to foster home confinement^41^ and internet-associated risky behaviors^42^. Sayeed and colleagues^43^ identified several risk factors for the development of PFU during the Covid-19 pandemic, such as having relationship break-ups or a history of violence and stressful life events.

However, it is worth noting that no prior longitudinal studies examined the relationship between patterns of PFU, well-being and distress during the second wave of the COVID-19 pandemic.

The present longitudinal study examined (i) whether distinct patterns of PFU could be identified through LCA on the basis of the following indicators: Facebook passive use, social comparison on Facebook, FoMO, and numbers of Facebook friends; and (ii) whether both moment-related evaluations, as well as changes in both psychological distress (i.e. depressive symptoms and Fear of COVID-19 pandemic) and well-being, differ among people with different patterns of PFU. According to prior evidence regarding positive associations between PFU and psychological distress^2,4,19,29,44,45^, it was hypothesized that the group with a higher dysfunctional pattern of PFU would have greater psychological distress and lower well-being across different stages of the second wave of pandemic.

## Methods

### Participants and procedure

Participants comprised college students at two large Universities in southern Italy. Four hundred and ninety-four participants consented to participate and completed an online survey at T0 (October 2020, a pandemic time in which new restrictions were implemented because of the spread of the second wave of COVID-19). Of these, two hundred and twenty-four participants (response rate 45.3%) completed the survey at T1 (December 2020, a period characterized by tightened containment measures and “red zones” for the Christmas holidays) and one hundred and ninety-one participants (response rate 38.7%) completed the survey at T2 (February 2021; a period characterized by the easing of restrictions). One participant was identified as a univariate outlier and was subsequently excluded from the analyses (see the Results section), thus 493 (80.3% females; M_age_ = 24.55 ± 7.25; age range = 18–63), 224 (78.1% females; M_age_ = 24.49 ± 6.61; age range = 18–57) and 190 (82.1% females; M_age_ = 25.00 ± 6.97; age range = 18–57) participants were considered for the three waves, respectively. Since we kept missing data points when matching the data for the three waves, the analytical sample included 493 participants. They were recruited through an announcement in the University and via on-line advertisements. Participation was voluntary and participants received no compensation. Information about the objectives of the study was given to the participants, and a prior statement of informed consent to participate was obtained from each participant. The online questionnaire took approximately 15–20 min to be completed. The research was conducted in accordance with the ethical standards of the Italian Psychological Association (AIP), as well as the Declaration of Helsinki. Participants’ demographic information and health-related data, as reported in Table 1.

**Table 1:** Participants’ demographic information and health-related data.

Variable	Participants (*N* = 493)
Age, *M* (SD)	24.55 (7.25)
**Gender, *n (%)***	
Females	396 (80.3)
Males	97 (19.7)
**Educational level, *n (%)***	
8 years of education	12 (2.4)
13 years of education	265 (53.8)
Degree/post-degree	216 (43.8)
**Marital status, *n (%)***	
In a relationship/married	267 (54.2)
Single/divorced/widowed	226 (45.8)
Personal COVID-19 infection, n (%) yes	18 (3.7)
COVID-19 infection among relatives/friends, n (%) yes	154 (31.2)

### Measures

At each wave, the first part of the questionnaire was used to collect information about participants’ demographic characteristics, including age, gender, educational level and marital status. In the next part, some questions about personal and relatives’ experiences of COVID-19 infection were inserted (in order to describe participants’ exposure to the virus as well as to account for the relationship that these variables may have with the variables of the study, such as depression and fear of COVID-19)^46^. Finally, data about PFU (i.e., number of Facebook friends, social comparison orientation on Facebook, passive use of Facebook and FoMO), psychological distress (i.e., depressive symptoms and fear of COVID-19 pandemic) and well-being were collected.

#### Social comparison orientation on Facebook

The *Iowa-Netherlands Comparison Orientation Measure* (INCOM)^47^ is an 11-item self-report measure of social comparison orientation (e.g., “*I often compare myself with respect to what I have accomplished in life*”). For the purposes of the current study, the scale was adapted by asking participants to think about the social interactions and behavior that are established on Facebook (e.g., “*When I use Facebook, I often compare myself with respect to what I have accomplished in life*”). Items were rated on a 5-point Likert scale, from 1 (*Strongly Disagree*) to 5 (*Strongly Agree*) with higher overall scores indicating a greater Facebook Social comparison orientation^40^. The scale demonstrated good internal consistency in the present study at each time-point (Cronbach’s α = 0.84, 0.87 and 0.85 for T0, T1 and T2, respectively).

#### Passive use of Facebook

The *Active and Passive Facebook Use Scale* (APUF)^18^ is a 7-item self-report measure of passive use of Facebook. Participants were asked to rate the frequency of use for some Facebook activities (e.g. *“Reading posts”*) on a 7-point Likert scale, from 1 (*Never to*) to 7 (*More than once a day*) with higher overall scores indicating a greater passive use of Facebook. The scale demonstrated acceptable-to-good internal reliability in the present study at each time-point (Cronbach’s α = 0.81, 0.81 and 0.79 at T0, T1 and T2, respectively).

#### Fear of missing out

The *Fear of Missing Out scale* (FoMOs)^26,48^ is a 10-item self-report measure of fear of missing out (e.g. “*I get anxious when I don’t know what my friends are up to*”). Items were rated on a 5-point Likert scale, from 1 (*Not at all true of me*) to 5 (*Extremely true of me*) with higher overall scores indicating more severe fear of missing out. The scale demonstrated good internal consistency in the present study at each time-point (Cronbach’s α = 0.82, 0.84 and 0.84 at T0, T1 and T2, respectively).

#### Depressive symptoms

The 7-item Depression subscale (DASS-D) of the Italian adaptation of *Depression, Anxiety and Stress Scale* (DASS-21)^49,50^ was used to measure depressive symptoms (e.g.*“I felt down hearted and blue”*). Items were rated on a 4-point Likert scale, from 0 (*It's never happened to me*) to 3 (*It's happened to me most of the time*) with higher overall scores indicating more severe depression. The scale demonstrated excellent internal consistency in the present study at each time-point (Cronbach’s α = 0.91, 0.91 and 0.93 for T0, T1 and T2, respectively).

#### Fear of COVID-19 pandemic

The *Multidimensional Assessment of COVID-19-Related Fears* (MAC-RF)^45^ is an 8-item self-report measure of fear of COVID-19 pandemic (e.g., “*During the coronavirus pandemic I constantly feel that I have to do something*”). Items were rated on a 5-point Likert scale, from 0 (*Strongly Disagree*) to 4 (*Strongly Agree*) with higher overall scores indicating more severe fear of the COVID-19 pandemic. In the present study, the scale demonstrated acceptable-to-good internal consistency in the present study at each timepoint (Cronbach’s α = 0.75, 0.80 and 0.77 at T0, T1 and T2, respectively).

#### Well-being

The *Satisfaction with Life Scale* (SWLS)^51,52^ is a 5-item self-report measure of well-being (e.g., “*If I could live my life over, I would change almost nothing*”). Items were rated on a 5-point Likert scale, from 1 (*Strongly Disagree*) to 7 (*Strongly Agree*) with higher overall scores indicating a greater well-being. The scale demonstrated good-to-excellent internal reliability in the present study at each timepoint (Cronbach’s α = 0.88, 0.87 and 0.90 at T0, T1 and T2, respectively).

### Statistical analysis

Data analyses were conducted using IBM SPSS (v. 22), Mplus (v. 7.0) and HLM software (v. 8.2). As a preliminary step in the data analysis, attrition analysis was conducted in order to compare participants with complete data with those with missing data at T1 and/or T2. Cronbach’s alphas were computed for all scales in order to assess their internal consistency. The normality of continuous variables was checked examining their skewness and kurtosis values. Descriptive statistics (means and standard deviations for continuous variables and frequencies and percentages for categorical variables) were computed for demographics and variables of interest.

As a first step in the data analysis, Latent Class Analysis (LCA) was conducted in order to classify the participants into different groups according to their PFU (i.e., number of Facebook friends, Passive use of Facebook, Social Comparison Orientation on Facebook and FoMO) at T0. We ranked models containing the one to four latent class to find a more meaningful and parsimonious model. The following fit indicators were examined to determine how many groups should be classified: Bayesian information criterion (BIC), sample size adjusted BIC (aBIC), entropy, Lo-Mendell Rubin likelihood ratio test (LMR LRT), and bootstrap likelihood ratio test (BLRT). The most suitable model had the following fit indices: BIC and aBIC should be lower; entropy should be larger and LMR and BLRT should be significant^53^. Moreover, the clinical meaning of the latent classes was also considered when selecting the model.

As a second step in the data analysis, we tested for the presence of significant linear changes in psychological distress (i.e., depressive symptoms and fear of COVID-19 pandemic) and well-being, from baseline to 4-months later, using 2-level Hierarchical Linear Models (HLMs). HLMs are considered one of the best statistical techniques for examining longitudinal changes in nested data^54^. Then, we entered the classes of participants -obtained through LCAs- as predictors of the longitudinal changes in psychological distress and well-being. This allowed us to test whether participants in specific classes experienced different time slopes compared to those from other classes. In addition, we compared the levels of each dependent variable at T1 and T2 across the classes through HLMs, changing how time was coded in our models (i.e., for comparisons at T1, time was coded as “− 1”, “0” and “1” for the three time points, respectively; for comparisons at T2, it was coded as “− 2”, “− 1” and “0”) and testing for significant group differences at the Intercept.

Effect sizes indicating the proportion of within-person variance were accounted for by adding the linear parameter and were assessed and reported using pseudo-R^2^^55^. Their magnitude was interpreted according to guidelines (0.01 = small, 0.06 = medium, > 0.14 = large)^56^.

### Ethics

The study was conducted in accordance with the ethical standards of the Italian Psychological Association (AIP), as well as the Helsinki Declaration of 1975 and its later amendments. Informed consent was obtained from all individual participants to be included in the study.

### Institutional review board statement

The study was approved by the Ethics Committee of the University of Palermo (Ethic Committee approval code: 37/2021).

## Results

### Preliminary analyses

At the baseline, no significant differences on demographics (i.e., age, gender and marital status), health-related data (i.e., personal and relatives COVID-19 infection), PFU characteristics (i.e., social comparison on Facebook, Facebook passive use and FoMO), psychological distress (i.e., depressive symptoms and fear of COVID-19 pandemic) or well-being at T0 were found between participants with complete data on all waves and those with missing data at T1 and/or T2. Significant differences were found only for educational level and number of Facebook friends. The normality of continuous variables was checked, and a positive skewed distribution was found for the number of Facebook friends. One univariate outlier was removed, and square root transformation was conducted to improve the normality of this variable. All other variables revealed no substantial violation of normality regarding data distribution at each time point (|Sk|< 1; Ku range: − 1.273 to 1.393).

### Latent class analysis of Facebook users

LCA identified three classes of participants. Evaluating one to four class models, the three-class model revealed the best solution (Table 2). Class 3 (*n* = 143; 29%) had the highest scores on all PFU indicators; therefore, it was defined as the “High PFU users”. Class 2 (*n* = 28; 6%) had the lowest scores on all indicators; therefore, it was defined as the “Healthy users”. Finally, Class 1 (*n* = 322; 65%) had indicators’ scores between Class 3 and Class 2; therefore, it was defined as the “Moderate PFU users”. Descriptives across all time points are reported in Table 3 for the whole group and for the three classes, separately. Correlations among the study variables at T0 are reported in Supplementary Table S1 (Supplementary Materials).

**Table 2:** LCA model fit indices.

Model	BIC	aBIC	Entropy	LMR LRT	BLRT
#1	13,379.771	13,354.379	–	–	–
#2	13,210.069	13,168.807	0.722	− 6665.084***	− 6665.084***
#3	13,179.563	13,122.431	0.760	− 6564.731***	− 6564.731***
#4	13,182.823	13,109.821	0.790	− 6533.977	− 6533.977***

*LCA* latent class analysis, *BIC* Bayesian information criterion, *aBIC* sample size adjusted BIC, *LMR LRT* Lo-Mendell Rubin likelihood ratio test, *BLRT* bootstrap likelihood ratio test.

^#^Number of classes.

***p < .001.

**Table 3:** Means, standard deviations and total N for all variables, across all time points, for the entire sample and separately for each class.

Variable	Group	T0		T1		T2	
		*N*	Mean (SD)	*N*	Mean (SD)	*N*	Mean (SD)
Depressive symptoms	Total	490	9.13 (5.89)	224	8.53 (5.86)	189	9.42 (6.52)
	Healthy users	28	5.46 (5.02)	12	4.25 (4.99)	9	6.33 (6.69)
	Moderate PFU users	321	8.16 (5.55)	151	7.40 (5.46)	132	8.33 (5.99)
	High PFU users	141	12.09 (5.67)	61	12.18 (5.31)	48	12.98 (6.67)
Fear of COVID-19 pandemic	Total	493	15 (5.82)	223	14.77 (5.91)	190	13.86 (5.77)
	Healthy users	28	12.04 (6.48)	12	11.58 (6.46)	9	9.44 (6.41)
	Moderate PFU users	322	14.24 (5.39)	151	13.97 (5.54)	133	13.31 (5.42)
	High PFU users	143	17.29 (5.94)	60	17.42 (5.90)	48	16.23 (5.83)
Well-being	Total	491	21.37 (6.51)	224	21.28 (6.22)	189	22.01 (6.61)
	Healthy users	28	24.50 (5.72)	12	24.25 (5.24)	9	24.33 (5.79)
	Moderate PFU users	322	22.00 (6.49)	151	21.75 (6.31)	132	22.36 (6.54)
	High PFU users	141	19.3 (6.18)	61	19.54 (5.83)	48	20.63 (6.82)
Number of Facebook friends	Total	415	719.82 (622.67)	–	–	–	–
	Healthy users	17	231.24 (215.38)	–	–	–	–
	Moderate PFU users	278	690.94 (581.23)	–	–	–	–
	High PFU users	120	855.94 (708.19)	–	–	–	–
Passive use of Facebook	Total	487	32.69 (8.16)	223	31.80 (8.01)	188	31.80 (7.49)
	Healthy users	28	14.71 (5.89)	12	18.50 (6.19)	9	14.89 (6.51)
	Moderate PFU users	320	32.85 (6.52)	150	31.87 (6.43)	132	31.57 (5.96)
	High PFU users	139	35.92 (7.32)	61	34.26 (9.28)	47	35.68 (6.93)
Social comparison on Facebook	Total	483	23.82 (7.93)	221	23.40 (8.42)	189	22.92 (7.82)
	Healthy users	25	15.12 (4.41)	12	13.83 (3.13)	9	14.11 (3.22)
	Moderate PFU users	319	20.63 (5.28)	150	21.29 (6.96)	133	20.87 (6.24)
	High PFU users	139	32.71 (5.94)	59	30.71 (7.60)	47	30.43 (7.18)
Fear of missing out	Total	493	23.88 (7.40)	223	23.54 (7.51)	190	23.70 (7.32)
	Healthy users	28	14.82 (3.63)	12	16.00 (3.10)	9	15.67 (3.46)
	Moderate PFU users	322	21.08 (5.25)	151	21.34 (6.29)	133	21.71 (6.09)
	High PFU users	143	31.95 (5.03)	60	30.60 (5.91)	48	30.71 (5.96)

*PFU* problematic Facebook use, *T0* October, 2020, *T1* December, 2020; *T2* February, 2021.

### Longitudinal changes in psychological distress and well-being across the three classes

The 2-level HLMs models evidenced non-significant longitudinal changes in measures of psychological distress and well-being, from baseline to 4-months later. That is to say, participants from the entire sample did not experience significant changes in psychological distress nor in well-being over time. The addition of the predictor "Time" at level-1 of all models accounted for 17–33% of the within-patient variance in the dependent variables, with large effects (see Table 4). Moreover, the between-person level accounted for most of the variability in all the dependent variables (range: 0.71–0.78).

**Table 4:** Fixed effects for the longitudinal changes in psychological distress (i.e. depressive symptoms and fear of COVID-19 pandemic) and well-being from baseline to 4 months later in the full sample of participants (n = 493).

Variable	β_10_	*SE*	*t*-value	*df*	*p*-value	*R*^*2*^	Within-person variance	Between-person variance
Depressive symptoms	0.20	0.17	1.167	489	0.25	0.33	0.29	0.71
Fear of COVID-19 pandemic	− 0.21	0.15	− 1.394	492	0.19	0.17	0.27	0.73
Well-being	0.29	0.16	1.858	490	0.064	0.19	0.22	0.78

R^2^ refers to pseudo-R^2^ indicating the proportion of within-person variance accounted for by adding the “Time” parameters to the model.

*SE* standard error of the regression coefficient, *df* degrees of freedom.

We then added the dummy-coded grouping variables as second-level predictors in our models: at baseline (T0), participants from the three groups reported significantly different levels (all *p*s < 0.05) in psychological distress (High PFU users > Moderate PFU users > Healthy users) and life satisfaction (High PFU users < Moderate PFU users < Healthy users; see Table 3 for descriptives and Supplementary Table S2 for *t*- and *p*-values). The only non-significant comparison was that between “Healthy users” and “Moderate PFU users” for fear of COVID-19. Interestingly, the grouping variable did not predict the longitudinal changes over time in psychological distress and well-being. That is to say, individuals clustered on the basis of their PFU pattern reported significantly different levels of psychological distress and well-being at baseline, which remained consistent over time (i.e., did not change). We further compared the levels of each dependent variable at T1 and T2 across the three classes; at two months follow-up, High PFU users reported greater levels of psychological distress and lower levels of well-being than the other two classes. Similarly, Moderate PFU users reported greater distress than Healthy users, but differences on well-being were not significant. At four months follow-up, High PFU users reported greater levels of psychological distress and lower levels of well-being than individuals belonging to the other two classes. Furthermore, Moderate PFU users reported greater fear of the COVID-19 pandemic than Healthy users, but all other comparisons were non-significant (see Table 3 for descriptives and Supplementary Tables S3 and S4 for *t*- and *p*-values).

Analyses re-ran controlling for personal COVID-19 infection and COVID-19 infection among relatives/friends at each time point (i.e., as time-varying covariates) led to almost identical results. Thus, our findings were robust in regard to the effect of these potentially confounding variables (see Supplementary Table S5).

## Discussion

The current study showed that a three-class model categorized effectively adults presenting different degrees of problematic Facebook use. The “High PFU users” reported greater passive use of Facebook, higher tendency toward online social comparison on Facebook, a greater number of online friends, and higher levels of FoMO. Healthy users showed the lowest scores on all characteristics of PFU, whereas participants in the “Moderate PFU users” reported mild scores on PFU variables ranging between the High PFU and Healthy users.

Consistently with our hypothesis (i.e., the class with higher level of PFU would have significantly greater psychological distress and lower well-being across the three waves), the findings of the study showed that participants with higher PFU also showed higher levels of psychological distress (i.e., depressive symptoms and fear of COVID-19) as well as lower well-being at each time point. These findings support previous evidence regarding the association between PFU and psychological distress and the negative link between life satisfaction and PFU^6^. Moreover, the current study adds preliminary evidence that passive Facebook use, online social comparison and FoMO may represent core characteristics of PFU. Prior studies suggested that passive social media use was associated with social comparison, which in turn predicted levels of stress during the pandemic^22^. It was suggested that passive social media use can negatively affect well-being due to social comparison with those better off than oneself as well as feelings of envy^21^. Conversely, individuals who report less problematic use of social media (with lower levels of passive use and lower online social comparison) may be less exposed to others' online content^31^. This may partially account for the higher scores for subjective well-being of users in the Healthy and Moderate PFU classes. The current findings further support the role of FoMO as an important facet of the individual’s impaired control with social media and as a correlate of psychological distress^26,57,58^.

During the second wave of the COVID-19 pandemic, it is also likely that the ongoing social restrictions may have increased PFU (e.g., through increased passive exposure) and worsened subjective well-being^59^, which exacerbated the use of SNSs for those who had already been problematic users before the pandemic. These findings may also explain the results regarding significantly lower levels of distress among both the Moderate PFU users and Healthy users during the second wave of the pandemic.

Generally, social media-related activities may have been a major channel in the search for COVID-19-related information during the second wave of the pandemic^60^. Therefore, a vicious cycle may have been generated and subsequently a positive relationship with the PFU^61^. This leads to the speculation that people with more severe PFU may be more exposed to COVID-19 relevant information, and it may then result in exaggerated psychological distress^62^.

The findings of the present study further indicated that the trajectory of psychological distress (i.e., depressive symptoms and fear of COVID-19) and well-being across three stages of the second wave of pandemic remained stable for all the three classes. Thus, participants clustered as “High PFU users” reported significantly higher levels of depressive symptoms and fear of COVID-19 as well as lower levels of well-being, which remained consistent over time. Our results may suggest that the association between the severity of PFU, psychological distress and well-being may be related to stable trait-like and time-invariant differences between individuals (between-person variance) rather than state-like changes (within-person variance) which commonly refers to those that occur from one assessment point to the next one^32^.

The current study extends our understanding of how PFU is associated with psychological distress and well-being by using a longitudinal design. Proposed implications are especially valuable when the relationship between COVID-19 pandemic, Facebook use, psychological distress and well-being is addressed. In addition, the results of the present study also included a period characterized by the easing of restrictions, which emphasized the importance of the potential need to maintain good mental health, even after the pandemic is over. However, some limitations should be considered when interpreting results. Firstly, the results may not be generalized to other countries due to discrepancies in the stage of COVID-19 infection and different governments’ policies aimed at limiting the spread of the virus. Secondly, the assessment of a non-stratified population with different recruitment procedures does not make these data generalizable. Thirdly, the self-report assessment may also limit conclusions from these results because the accuracy of the participants’ answers might have been affected. Future research needs to use a stratified sample whilst adding objective assessments of PFU.

## Conclusion

These results underlined the link between problematic Facebook use during the COVID-19 pandemic, psychological distress and well-being. It should be emphasized that increased time spent on social media was unavoidable during the pandemic when many activities were suspended. Therefore, in interpreting these results, one must be aware that, regardless of people’s usual online habits, the utilization of social media and information acquired through online activities may have triggered the onset of PFU, on top of general, increased internet use during the pandemic.

## Supplementary Information

Supplementary Information.

## Data Availability

The dataset generated for this study are available on request from the corresponding author.
