# Supplementary material for: A longitudinal investigation on problematic Facebook use, psychological distress and well-being during the second wave of COVID-19 pandemic
Source: Sci Rep. 2022 Dec 17;12:21828. doi: 10.1038/s41598-022-26281-0 (PMC9758466; doi:10.1038/s41598-022-26281-0)
Supplement: Supplementary file 1 — Supplementary Information. [file 41598_2022_26281_MOESM1_ESM.docx]

**Supplementary Materials**

Supplementary Table S1

*Correlations among the study variables at T0, for the whole group and separately for the three classes*

| Group |  | 1. | 2. | 3. | 4. | 5. | 6. |
| --- | --- | --- | --- | --- | --- | --- | --- |
| Total | 1. Number of Facebook Friends | - |  |  |  |  |  |
|  | 2. Passive Use of Facebook | .308^**^ | - |  |  |  |  |
|  | 3. Social Comparison Orientation on Facebook | .094 | .290^**^ | - |  |  |  |
|  | 4. Fear of Missing Out | .096 | .285^**^ | .503^**^ | - |  |  |
|  | 5. Depressive symptoms | -.081 | .090^*^ | .257^**^ | .438^**^ | - |  |
|  | 6. Well-being | .059 | -.121^**^ | -.162^**^ | -.295^**^ | -.538^**^ | - |
|  | 7. Fear of COVID-19 pandemic | .039 | .138^**^ | .242^**^ | .341^**^ | .374^**^ | -.115^*^ |
| Healthy Users | 1. Number of Facebook Friends | - |  |  |  |  |  |
|  | 2. Passive Use of Facebook | -.192 | - |  |  |  |  |
|  | 3. Social Comparison Orientation on Facebook | -.144 | -.187 | - |  |  |  |
|  | 4. Fear of Missing Out | .421 | -.245 | .181 | - |  |  |
|  | 5. Depressive symptoms | -.149 | -.084 | -.028 | .508^**^ | - |  |
|  | 6. Well-being | .034 | .136 | -.167 | -.363 | -.510^**^ | - |
|  | 7. Fear of COVID-19 pandemic | -.064 | .097 | -.061 | .207 | .291 | -.020 |
| Moderate PFU Users | 1. Number of Facebook Friends | -- |  |  |  |  |  |
|  | 2. Passive Use of Facebook | .181^**^ | - |  |  |  |  |
|  | 3. Social Comparison Orientation on Facebook | -.014 | .116^*^ | - |  |  |  |
|  | 4. Fear of Missing Out | -.097 | .017 | -.056 | - |  |  |
|  | 5. Depressive symptoms | -.156^**^ | -.002 | .039 | .276^**^ | - |  |
|  | 6. Well-being | .143^*^ | -.072 | -.031 | -.212^**^ | -.523^**^ | - |
|  | 7. Fear of COVID-19 pandemic | -.058 | .086 | .046 | .234^**^ | .269^**^ | -.116^*^ |
| High PFU Users | 1. Number of Facebook Friends | - |  |  |  |  |  |
|  | 2. Passive Use of Facebook | .352^**^ | - |  |  |  |  |
|  | 3. Social Comparison Orientation on Facebook | -.053 | .015 | - |  |  |  |
|  | 4. Fear of Missing Out | .054 | .052 | -.042 | - |  |  |
|  | 5. Depressive symptoms | -.173 | -.165 | -.023 | .333^**^ | - |  |
|  | 6. Well-being | .059 | .035 | .096 | -.162 | -.465^**^ | - |
|  | 7. Fear of COVID-19 pandemic | .078 | -.070 | .129 | .208^*^ | .408^**^ | .057 |

*Note.* PFU = Problematic Facebook Use; * *p* < .05; ** *p* < .01

Supplementary Table S2

*B, standard errors, degrees of freedom, t- and p-values for the between-group comparisons at T0 on all dependent variables (Depressive Symptoms, Well-being, Fear of COVID-19 pandemic)*

| T0 – Depressive Symptoms | β_10_ | *SE* | *t*-value | *df* | *p*-value |
| --- | --- | --- | --- | --- | --- |
| Healthy Users *vs* High PFU Users | -6.692 | 1.033 | -6.478 | 487 | < .001 |
| Moderate PFU Users *vs* High PFU Users | -3.895 | 0.559 | -6.966 | 487 | < .001 |
| Healthy Users *vs* Moderate PFU Users | 2.797 | 0.972 | -2.877 | 487 | .004 |
| T0 – Fear of COVID-19 pandemic |  |  |  |  |  |
| Healthy Users *vs* High PFU Users | -5.233 | 1.281 | -4.085 | 490 | < .001 |
| Moderate PFU Users *vs* High PFU Users | -3.081 | 0.582 | -5.291 | 490 | < .001 |
| Healthy Users *vs* Moderate PFU Users | -2.152 | 1.217 | -1.767 | 490 | .078 |
| T0 – Well-Being |  |  |  |  |  |
| Healthy Users *vs* High PFU Users | 5.158 | 1.171 | 4.405 | 488 | < .001 |
| Moderate PFU Users *vs* High PFU Users | 2.633 | 0.624 | 4.221 | 488 | < .001 |
| Healthy Users *vs* Moderate PFU Users | 2.526 | 1.113 | 2.270 | 488 | .024 |

Supplementary Table S3

*B, standard errors, degrees of freedom, t- and p-values for the between-group comparisons at T1 on all dependent variables (Depressive Symptoms, Well-being, Fear of COVID-19 pandemic)*

| T1 – Depressive Symptoms | β_10_ | *SE* | *t*-value | *df* | *p*-value |
| --- | --- | --- | --- | --- | --- |
| Healthy Users *vs* High PFU Users | -6.840 | 1.182 | -5.787 | 487 | < .001 |
| Moderate PFU Users *vs* High PFU Users | -4.226 | 0.582 | -7.258 | 487 | < .001 |
| Healthy Users *vs* Moderate PFU Users | -2.614 | 1.121 | -2.333 | 487 | .020 |
| T1 – Fear of COVID-19 pandemic |  |  |  |  |  |
| Healthy Users *vs* High PFU Users | -5.780 | 1.269 | -4.554 | 490 | < .001 |
| Moderate PFU Users *vs* High PFU Users | -2.845 | 0.565 | -5.031 | 490 | < .001 |
| Healthy Users *vs* Moderate PFU Users | -2.936 | 1.213 | -2.421 | 490 | .016 |
| T1 – Well-Being |  |  |  |  |  |
| Healthy Users *vs* High PFU Users | 4.364 | 1.146 | 3.808 | 488 | < .001 |
| Moderate PFU Users *vs* High PFU Users | 2.586 | 0.654 | 3.956 | 488 | < .001 |
| Healthy Users *vs* Moderate PFU Users | 1.778 | 1.069 | 1.663 | 488 | .097 |

Supplementary Table S4

*B, standard errors, degrees of freedom, t- and p-values for the between-group comparisons at T2 on all dependent variables (Depressive Symptoms, Well-being, Fear of COVID-19 pandemic)*

| T2 – Depressive Symptoms | β_10_ | *SE* | *t*-value | *df* | *p*-value |
| --- | --- | --- | --- | --- | --- |
| Healthy Users *vs* High PFU Users | -6.988 | 1.660 | -4.210 | 487 | < .001 |
| Moderate PFU Users *vs* High PFU Users | -4.557 | 0.813 | -5.601 | 487 | < .001 |
| Healthy Users *vs* Moderate PFU Users | -2.432 | 1.575 | -1.543 | 487 | .123 |
| T2 – Fear of COVID-19 pandemic |  |  |  |  |  |
| Healthy Users *vs* High PFU Users | -6.328 | 1.779 | -3.557 | 490 | < .001 |
| Moderate PFU Users *vs* High PFU Users | -2.608 | 0.723 | -3.607 | 490 | < .001 |
| Healthy Users *vs* Moderate PFU Users | -3.720 | 1.718 | -2.165 | 490 | .031 |
| T2 – Well-Being |  |  |  |  |  |
| Healthy Users *vs* High PFU Users | 3.571 | 1.592 | 2.243 | 488 | .025 |
| Moderate PFU Users *vs* High PFU Users | 2.539 | 0.844 | 3.008 | 488 | .003 |
| Healthy Users *vs* Moderate PFU Users | 1.031 | 1.493 | 0.691 | 488 | .490 |

Supplementary Table S5

*Fixed effects for the longitudinal changes in psychological distress (i.e. depressive symptoms and fear of COVID-19 pandemic) and well-being from baseline to 4 months later in the full sample of participants, while controlling for personal COVID-19 infection and COVID-19 infection among relatives/friends during all time points.*

| Variable | β_10_ | *SE* | *t*-value | *df* | *p*-value | *R^2^* |
| --- | --- | --- | --- | --- | --- | --- |
| Depressive symptoms | 0.24 | 0.19 | 1.284 | 488 | .20 | .02 |
| Fear of COVID-19 pandemic | -0.28 | 0.15 | -1.824 | 491 | .069 | < .01 |
| Well-being | 0.28 | 0.17 | 1.636 | 489 | .10 | < .01 |

*Note. N = 493.* R^2^ refers to pseudo-R^2^ indicating the proportion of within-person variance accounted for by adding the time-varying covariates (Personal COVID-19 infection and COVID-19 infection among relatives/friends) to the unconditional model with “Time” as only predictor of the dependent variable; SE = standard error of the regression coefficient; df = degrees of freedom. Personal COVID-19 infection and COVID-19 infection among relatives/friends were entered as a dichotomous (0 = no; 1 = yes), group-centered level-1 predictor of each multilevel model.
